# Prevalence and Risk Factors of Type 2 Diabetes Mellitus Among Hepatitis B Virus Patients: A Large Retrospective Cohort Study

**DOI:** 10.7150/ijms.104839

**Published:** 2025-01-13

**Authors:** Fadi Abu Baker, Abdel-Rauf Zeina, Randa Taher Natour, Saif Abu Mouch, Yael Kopelman, Oren Shibolet, Ariel Israel

**Affiliations:** 1 Department of Gastroenterology and Hepatology, Hillel Yaffe Medical Center, Hadera, Israel. Affiliated to the Technion Faculty of Medicine, Haifa, Israel.; 2 Department of Radiology, Hillel Yaffe Medical Center, Hadera, Israel. Affiliated to the Technion Faculty of Medicine, Haifa, Israel.; 3 Department of Internal Medicine B, Hillel Yaffe Medical Center, Hadera, Israel. Affiliated to the Technion Faculty of Medicine, Haifa, Israel.; 4 Department of Gastroenterology and Hepatology, Tel-Aviv Medical Center, Tel-Aviv, Israel. Sackler Faculty of Medicine, Tel-Aviv University, Tel-Aviv, Israel.; 5 Research Institute, Leumit Health Services; Department of Epidemiology and Preventive Medicine, School of Public Health, Faculty of Medical & Health Sciences, Tel-Aviv University, Tel-Aviv, Israel.

**Keywords:** Hepatitis B virus, type 2 diabetes mellitus, screening, prevalence, risk factors

## Abstract

**Background:** Hepatitis B virus (HBV) infection and type 2 diabetes mellitus (T2DM) are both major health burdens worldwide. There is a suspected link between the two conditions, but the nature of the relationship is not well understood. This study aimed to investigate the prevalence of T2DM in patients with HBV, compared to matched non-HBV patients.

**Methods:** A retrospective cohort study was conducted using data from the Leumit-Health-Service (LHS) database, a major health insurance provider in Israel, covering the years 2000-2020. The study included patients with HBV infection and matched controls using propensity score matching. T2DM's prevalence was assessed and compared between both groups.

**Results:** A total of 3,436 HBV carriers and 34,360 matched controls were included. T2DM's prevalence was significantly higher among HBV patients compared to the control group (22.1% vs. 19.5%; P<0.001). Through multivariate analysis, we identified age, obesity, smoking, and specific HBV-related parameters, such as chronic active disease or evidence of advanced fibrosis at presentation, as independent risk factors for T2DM in HBV patients.

**Conclusions:** This study revealed a higher prevalence of T2DM in HBV patients compared to controls, and identified specific risk factors associated with T2DM in HBV patients. Enhanced screening and management of metabolic risk factors should be considered in this population.

## Introduction

T2DM and HBV infection pose significant challenges to public health, substantially impacting global morbidity and mortality 1,2. In addition, chronic HBV infection (CHB) is a major contributor to chronic hepatitis, cirrhosis, and hepatocellular carcinoma (HCC) 3. Simultaneously, the worldwide prevalence of T2DM and metabolic syndrome is rising, imposing substantial medical, economic, and social burdens 4.

Accumulating evidence suggests a possible association between chronic liver diseases and T2DM. Notably, chronic hepatitis C (HCV) infection consistently exhibits an elevated risk of T2DM 5-7. Existing medical literature indicates a common occurrence of moderate-to-advanced fibrosis among individuals with diabetes, which serves as a significant risk factor for the development of cirrhosis and increased overall mortality 8. Furthermore, growing evidence shows that T2DM may exacerbate complications associated with chronic HBV infection, such as liver fibrosis, cirrhosis, and HCC.

Exploring the risk factors for T2DM development and assessing its prevalence in individuals with chronic HBV infection has generated significant interest 9. While some clinical studies support the association between HBV infection and T2DM, others present contrary perspectives, suggesting a complex and multifactorial relationship 10-15. Moreover, recent research has provided evidence supporting a potential direct impact of HBV infection on glucose metabolism, contributing to the development of T2DM in infected individuals 16.

Many studies reporting a significantly higher risk of T2DM development in HBV-infected individuals fail to account for crucial demographic and anthropometric factors, such as age, sex, ethnicity, and body mass index (BMI), which can influence the prevalence of both T2DM and HBV infection.

Understanding the association between HBV infection and T2DM bears important clinical implications for managing HBV-infected individuals and preventing and treating T2DM. Therefore, this study aims to investigate the prevalence of T2DM in HBV-infected individuals compared to age-, sex-, ethnicity-, and BMI-matched non-HBV-infected individuals using a matching strategy within a large cohort of HBV patients.

## Materials and Methods

### Study design and data source

This retrospective cohort study aimed to investigate the prevalence of T2DM in patients with HBV compared to age-, sex-, ethnicity-, and body mass index (BMI)-matched non-HBV patients. To ensure robust comparisons, we used a 1:10 matching ratio, selecting ten non-HBV controls for each HBV patient from the same database. This approach maximized statistical power while maintaining balance between groups.

All the data utilized in this study were obtained from the computerized database of Leumit Healthcare Services (LHS), a large health maintenance organization (HMO) in Israel. As per the Israeli 1995 national health insurance law, each citizen is granted the entitlement to select any health fund, irrespective of age, salary, or health condition, ensuring access to all services encompassed within the national health package. In light of this, the population of LHS members is nationally representative. The data retrieved for analysis covered the period from 2000 to 2020, providing a substantial timeframe for examination. The LHS database is continuously updating and contains comprehensive information, including patients' demographics, physician diagnoses, medical procedures, hospitalizations, and a comprehensive record of all prescription medication dispensations and laboratory tests. The database is known for its high member retention rate and utilizes automatic search formulas with a comprehensive cross-validation approach to ensure data accuracy, rather than solely relying on active reporting by physicians. Therefore, the data obtained from LHS serve as a reliable and representative source for this study.

### Study population and selection criteria

The study included patients with HBV based on International Classification of Diseases, Ninth Revision, Clinical Modification (ICD-9-CM) codes and positive hepatitis B surface antigen (HBsAg) serology. Clinical and laboratory data were used to determine HBeAg status, chronic HBV phase (determined according to the European Association for the Study of the Liver(EASL) guidelines based on ALT, viral load and fibrosis stage), concomitant liver disease, and HBV treatment status, whenever available. Chronic active hepatitis was defined in accordance with the guidelines provided by the EASL. This definition encompassed patients who were HBeAg positive or negative and exhibited evidence of viral load greater than 2000 copies/mL and elevated ALT levels or fibrosis stage F2 or higher determined by biopsy, fibroscan, or, when unavailable, by using a FIB-4 score greater than 2.65. Patients with evidence of chronic hepatitis C or D virus coinfection, or those with a diagnosis of liver cirrhosis at inclusion were excluded. The index date for the study cohort was the date of HBV diagnosis.

For each HBV patient, non-HBV participants in a 1:10 ratio were selected as matched controls using propensity score matching. The non-HBV patients were selected from the same LHS database, had no history of HBV infection at any time during the study period, and had no evidence of T2DM (based on ICD-9-CM codes) before the index date of the matched HBV patient. The index date for the non-HBV patients was the same as the index date for their matched HBV patient. It is crucial to emphasize that only incident cases of type T2DM that developed during the study period were included in the final analysis. This rigorous selection criterion was applied to mitigate the potential influence of preexisting conditions, ensuring that the study findings remain unaffected by confounding factors.

The overall prevalence of T2DM during the study period was assessed, and the diagnosis rate of T2DM was calculated in each age-category (<20, 20-60, and >60) in both groups. Moreover, we performed subgroup analysis in CHB patients with full documentation of data on CHB phase, treatment, and outcome, to identify predictors of T2DM development among CHB patients.

The study was conducted in accordance with the Helsinki Declaration and Rules of Good Clinical Practice. The LHS institutional review board approved the study (protocol code 0216-19-ASF). The need to obtain consent for the collection, analysis, and publication of data was waived by the LHS ethics committee. All data were de-identified to protect patient privacy.

### Statistical analysis

Continuous variables were presented as mean ± standard deviation, and categorical variables as percentages, unless otherwise stated. The total cohort was divided into 1:10 ratios (study and age-, sex-, ethnicity-, and BMI-matched control groups) using the Propensity Score Matching in R program version 3.3. The normal distribution of quantitative parameters was tested using the Kolmogorov-Smirnov test. The t-test and Fisher exact test for categorical parameters were used to compare the differences between the groups. Multiple regression models were employed using a stepwise approach to identify predictors associated with the development of T2DMin CHB patients in terms of odds ratio and 95% confidence interval (95%CI). The statistical analysis was performed using SPSS version 25. A significance level of P < 0.05 was considered significant.

## Results

### Study population and baseline characteristics

The study included 3436 patients diagnosed with HBV based on ICD-9 codes and serology. These patients were matched with 34360 controls based on age, sex, ethnicity, and BMI. The study followed patients for an average of 148±16 months. The mean age of the HBV patients was 55.74 ±14.70, and males comprised the majority (56.7%) of the group. Most patients (71%) were of Jewish ethnicity, followed by 22.6% Arabs and 6.4% other ethnicities, which reflected the background prevalence in the Israeli population. A total of 70 (2%) of HBV patients had positive HDV antibodies, all of whom had normal liver enzymes and no evidence of active HDV and thus were included in the final analysis.

The baseline characteristics of the HBV and control patients are presented in **Table 1**.

### Prevalence of T2DM in HBV patients

**Figure 1:**
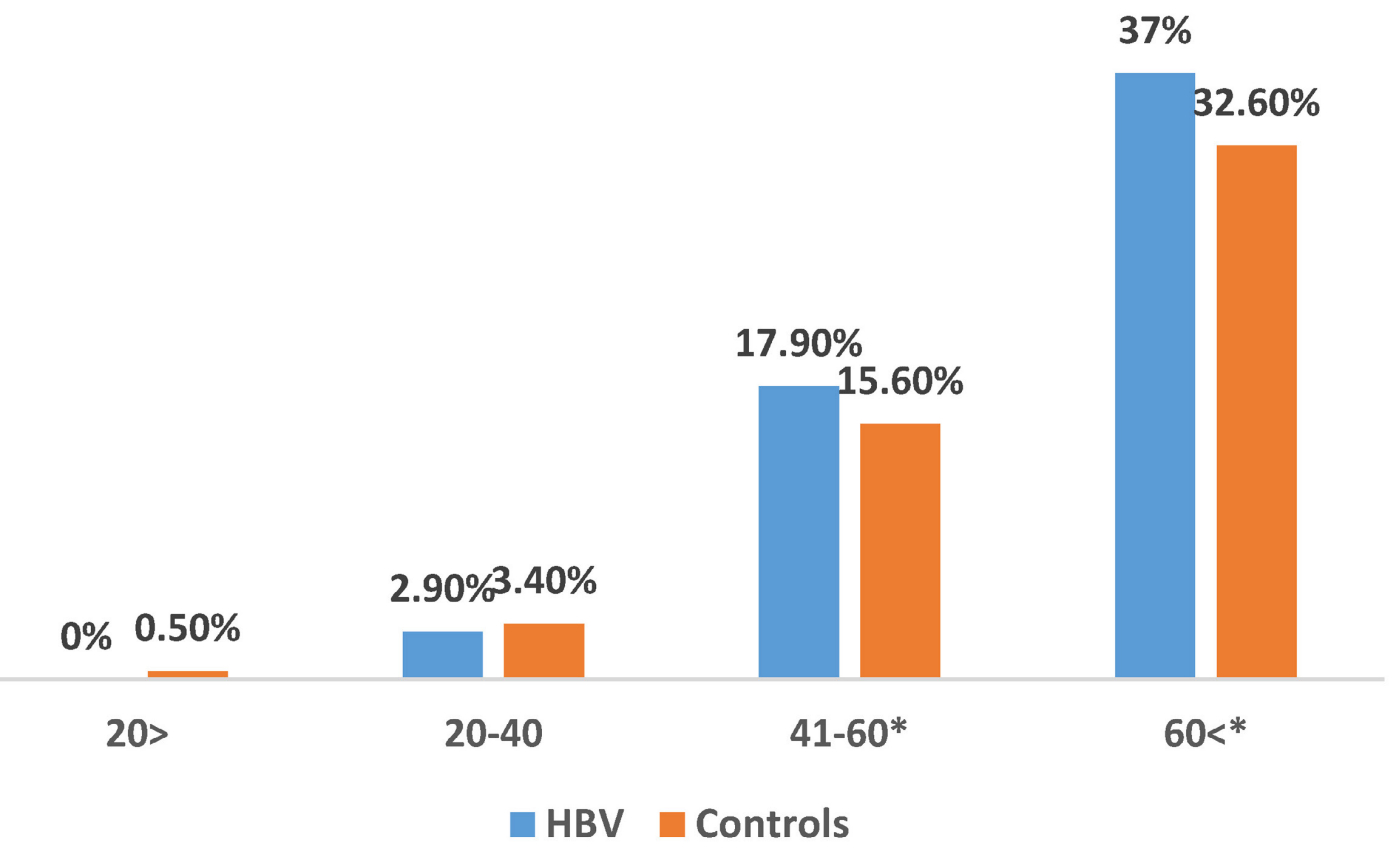
The prevalence of type 2 diabetes mellitus in HBV patients and matched controls across age groups. *Findings are clinically significant.

**Figure 2:**
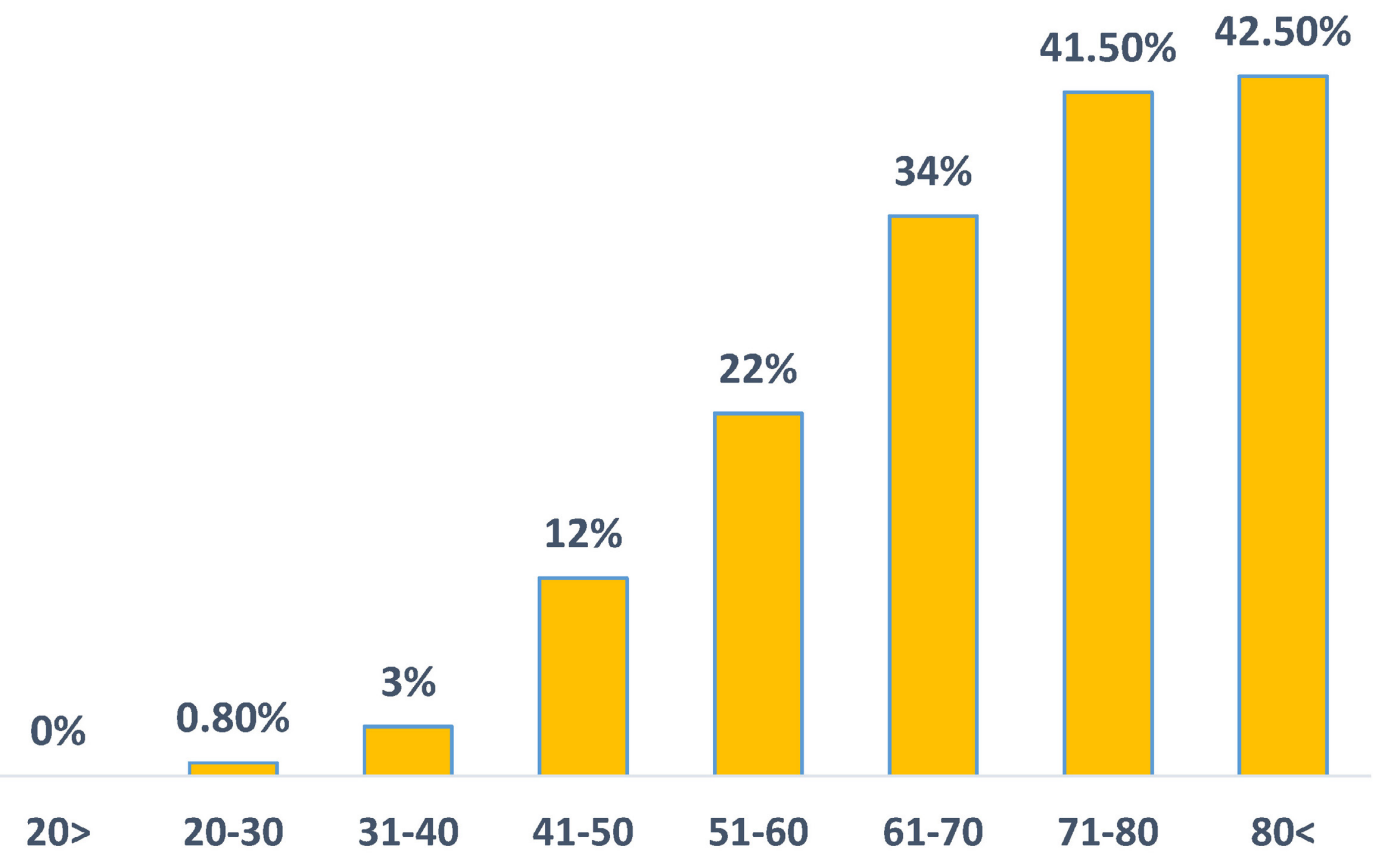
The prevalence of type 2 diabetes mellitus in HBV patients across age groups.

The prevalence of incident cases of T2DM during the study period was significantly higher in HBV patients compared to the control group (22.1% vs. 19.5%; P<0.001). Notably, a higher prevalence of T2DM was observed in HBV patients aged 40-60 (17.9% vs. 15.6%; P<0.01) and those above 60 (37% vs. 32.6%; P<0.001) (Figure 1). We show a linear increase in T2DM prevalence with age in HBV patients (figure 2). The mean time of T2DM development after HBV diagnosis was 6.1±2.4 years.

### Predictors of T2DM development in CHB patients

We performed a detailed subgroup analysis on a cohort of 1186 CHB patients who had comprehensive documentation of clinical, serological, and other relevant data pertaining to their CHB infection profile and phase to identify predictors associated with the development of T2DM in this population. We compared patients with CHB and T2DM (HBV-T2DM) to those with CHB without T2DM (HBV controls) (Table 2). The HBV-T2DM patients were older compared to HBV-controls (mean age 49.17±9.8 vs. 37.7±10.1; P<0.001), had higher rates of obesity (46.5% vs. 22.5%; P<0.01) and a higher prevalence of active or past smoking (32.9% vs. 24%; P=0.04), but there were no significant differences in ethnicity and gender distribution between the two groups. Interestingly, the HBV-T2DM patients exhibited elevated levels of ferritin and hypertriglyceridemia compared to HBV-controls, whereas there was no significant difference in low-density lipoprotein (LDL) levels between the groups.

We found that the HBV-T2DM patients had a significantly higher proportion of chronic active hepatitis compared to HBV controls (either HBeAg positive or negative) (48.8% vs. 40.1%; P=0.006) as was the prevalence of FIB-4 >2.65 at diagnosis, indicating advanced liver fibrosis (12.4% vs. 6.1%; P=0.02). The difference in the proportion of patients receiving nucleoside/tide analogs treatment between the groups was not statistically significant (p=0.09).

Multivariate analysis (Table 3) identified several independent predictors of T2DM development in CHB patients. Age (OR 1.06, 95% CI 1.04-1.08; P<0.01), obesity (OR=2.63, 95% CI 1.7-4.1; P<0.01), smoking (OR= 1.48, 95% CI 1.08-2.1; P=0.037), hypertriglyceridemia (OR= 2.88, 95% CI 2.2-4.1; P<0.01), chronic active hepatitis (OR=2.28, CI 1.2-4.3; P<0.01), and evidence of advanced fibrosis indicated by FIB-4>2.65 (OR=1.83, 95% CI 1.1-3.7; P=0.02) were all found to be significant independent predictors of T2DM development in this population of CHB patients.

## Discussion

Our study aimed to investigate the prevalence of T2DMamong HBV patients. To the best of our knowledge, this is the first study to investigate the prevalence of T2DM in a large HBV cohort using a propensity score matching strategy. Our results indicated a significant increase in the prevalence of T2DM in the HBV patient population compared to the matched control group. This finding is consistent with a growing body of evidence suggesting that patients with HBV infection may be at an increased risk for developing T2DM.

A few population-based studies have reported that the incidence and prevalence of T2DM are significantly higher in patients with chronic HBV infection compared to the general population 17,18. Lie *et al.* found that Chinese participants with chronic HBV infection or HBsAg negative/anti-HBc positive serology had a higher risk of T2DM 19. Similarly, Hong *et al.* reported that HBV and HCV infections were associated with increased T2DM prevalence and HBV infection with the risk of incident T2DM 20. Furthermore, the pooled results of a meta-analysis that included 12 studies suggest that HBV-infected patients have a significantly higher risk of developing T2DM compared to controls 21.

It is worth noting that most of these studies were conducted in Asian populations and may have been limited by selection bias and inadequate adjustment for confounding factors. Moreover, some studies reported no association between HBV infection andT2DM. In contrast, the current study was conducted on a large nationwide cohort of Israeli patients with several ethnicities and controlling for major factors associated with T2DM risk.

Our study found that the prevalence of T2DM in the HBV patient population was significantly higher in those over 40 years compared to controls, which is consistent with the findings of other studies 22.

Our study extends beyond the well-established metabolic factors associated with T2DM, such as obesity and hypertriglyceridemia, by identifying viral factors, specifically active chronic hepatitis and evidence of advanced fibrosis at presentation as potential contributors to the pathophysiology of T2DM in CHB patients. The exact mechanisms underlying the association between HBV infection and T2DM are not fully elucidated, with most of the previous literature focusing on the relationship between HBV-related cirrhosis and T2DM. However, chronic inflammation induced by the HBV infection and the production of pro-inflammatory cytokines was shown to contribute to insulin resistance and impaired glucose metabolism 24, 25. Additionally, experimental studies have suggested that the hepatitis B X protein (HBx) may play a role in the development of insulin resistance by interfering with the hepatic insulin signaling pathway 26.

It is important to note that while our findings suggest an increased risk of T2DM among HBV patients, other factors such as lifestyle factors, genetics, and comorbidities may also contribute to the development of diabetes in this population. Notably, our study accounted for ethnicity in the propensity score matching to address potential disparities in the prevalence of HBV and metabolic diseases between Jewish and Arab populations in Israel. Previous research, including our own work, has highlighted significant differences in obesity, diabetes, and HBV prevalence between these groups, which may influence the outcomes of epidemiological studies 27. While these adjustments minimize the risk of confounding, it is important to acknowledge that cultural, socioeconomic, and genetic factors unique to each ethnic group may still contribute to variations in metabolic outcomes. Further exploration of these disparities in future studies will be essential to provide more targeted interventions.

The optimal strategies for managing T2DM in patients with CHB infection has yet to be established, and further research is needed in this area. Nevertheless, the elevated risk of T2DM among patients with HBV has significant implications for clinical practice. It underscores the need for regular screening and monitoring for T2DM in this population. Clinicians should be aware of the potential metabolic effects of HBV infection and strictly manage other metabolic risk factors. Moreover, lifestyle interventions like weight loss, physical activity, and dietary changes should be specifically addressed by physicians taking care of patients with HBV infection regardless of phase or viral activity.

Our study has several limitations. First, the cross-sectional design of the study limits our ability to establish a causal relationship between HBV and T2DM, as temporal associations cannot be determined. Second, while we used propensity score matching to control for key variables such as age, sex, BMI, and ethnicity, other potential confounders, such as lifestyle habits, dietary patterns, physical activity levels, and family history of T2DM, were not available in the dataset and could not be accounted for. Third, the data were derived exclusively from LHS, the smallest health maintenance organization in Israel. While the LHS database provides comprehensive and high-quality data, its relatively small size compared to other HMOs in the country may limit the generalizability of our findings to the broader Israeli population.

## Conclusions

In conclusion, our study adds to the existing body of evidence supporting an association between HBV infection and an elevated risk of developing T2DM, particularly in individuals aged 40 and above. Additionally, we identified specific risk factors associated with T2DM development in this population. The findings should encourage physicians to monitor for early signs of disturbed glucose metabolism and manage T2DM risk factors. Further research is necessary to confirm these findings and better understand the underlying mechanisms that govern the interaction between HBV and T2DM.

## Figures and Tables

**Table 1:** Baseline characteristics of the HBV patients and matched controls.

Group	HBV (n=3436)	Control (n=34360)	P value
Age (mean±SD)	55.62±14.79	55.74 ±14.70	0.65
Sex (male) n/%	19,800 (57.6 %)	1,980 (57.6 %)	0.95
Ethnicity (Jewish) n/%	24,370 (70.9 %)	2,437 (70.9 %)	0.88
BMI (mean±SD)	27.44±4.92	27.34±5.00	0.25
Normal (BMI 18.5-25)	12,200 (35.5 %)	1,220 (35.5 %)	0.99
Underweight (BMI 16-18.5)	68 (1.87 %)	680 (1.88 %)	1
Overweight (BMI 25-30)	12,260 (35.7 %)	1,226 (35.7 %)	1
Obesity (BMI>30) n/%	9220 (26.8%)	922 (26.8%)	1

**Table 2:** Baseline characteristics and HBV infection profile in diabetic (HBV-T2DM) and non-diabetic (HBV- NoT2DM) chronic HBV patients.

Group	HBV- controls	HBV-T2DM	p-value
**Patients baseline characteristics**			
Age mean± SD	37.7±10.1	49.17± 9.8	P<0.001
Age<40	554 (58.6%)	56 (23.2%)	
Age 40-60	337 (35.7%)	146 (60.6%)	
Age> 60	54 (5.7%)	39 (16.2%)	
Gender (male)	397 (42%)	102 (41.5%)	P=0.30
Jewish ethnicity n (%)	601 (63.6%)	164 (68.0%)	P=0.20
Obesity (BMI >30)	214 (22.6%)	112 (46.5%)	P<0.001
Ferritin (ng/mL) Ferritin>200 (ng/mL)	101.41±69 91 (12.5%)	147.7±77 44 (21.9%)	P=0.014
Low-density lipoprotein (mg/dL)	110±34	113±35	P=0.093
Triglycerides (mg/dL) Triglycerides >150 (mg/dL)	113±66 438 (46.6%)	213±64 157 (65.1%)	P<0.001
Active/past smoker	227 (24%)	79 (32.9%)	P=0.044
Creatinine (mg/dL)	1±0.43	0.95±0.33	P=0.98
Chronic kidney disease (GFR<60)	12 (1.3%)	3 (1.2%)	P=1.0
**Chronic HBV profile**			
HBeAg status (negative)	823 (87.1%)	214 (88.9%)	P=0.12
Chronic Active hepatitis (HBeAg +/-)	379 (40.1%)	119 (48.8%)	P=0.006
FIB-4 >2.65	58 (6.1%)	30 (12.4%)	P=0.02
Nucleoside/tide analogs treatment	301 (31.9%)	83 (35.4%)	P=0.09

**Table 3:** Multivariate analysis of independent predictors for type 2 diabetes mellitus development in Hepatitis B Patients.

Variable	Odds Ratio	95% Confidence Interval	P-value
Age (per year increase)	1.06	1.04-1.08	<0.01
Obesity (BMI >30)	2.63	1.70-4.10	<0.01
Smoking	1.48	1.08-2.10	0.037
Hypertriglyceridemia	2.88	2.20-4.10	<0.01
Chronic active hepatitis	2.28	1.20-4.30	<0.01
Advanced fibrosis (FIB-4>2.65)	1.83	1.10-3.70	0.02
Ethnicity (non-Jewish)	1.44	0.92-2.11	0.22
Sex (male)	1.26	0.92-1.43	0.12

## Abbreviations

ALT: Alanine aminotransferase
anti-HBc: Hepatitis B core antibody
BMI: Body mass index
CHB: Chronic hepatitis B
HBsAg: Hepatitis B surface antigen
HBV: Hepatitis B virus
HBx: Hepatitis B X protein
HCC: Hepatocellular carcinoma
HCV: Hepatitis C virus
DM: Diabetes mellitus
T2DM: Type 2 diabetes mellitus
